# The Bionic High-Cushioning Midsole of Shoes Inspired by Functional Characteristics of Ostrich Foot

**DOI:** 10.3390/bioengineering10010001

**Published:** 2022-12-20

**Authors:** Rui Zhang, Liangliang Zhao, Qingrui Kong, Guolong Yu, Haibin Yu, Jing Li, Wei-Hsun Tai

**Affiliations:** 1 Key Laboratory of Bionic Engineering, Ministry of Education, Jilin University, Changchun 130022, China; 2 School of Physical Education, Quanzhou Normal University, Quanzhou 362000, China

**Keywords:** ostrich toe pad, metatarsophalangeal joint, high cushioning, midsole of soles, bionic design

## Abstract

The sole is a key component of the interaction between foot and ground in daily activities, and its cushioning performance plays a crucial role in protecting the joints of lower limbs from impact injuries. Based on the excellent cushioning performance of the ostrich foot and inspired by the structure and material assembly features of the ostrich foot’s metatarsophalangeal skeletal–tendon and the ostrich toe pad–fascia, a functional bionic cushioning unit for the midsole (including the forefoot and heel) area of athletic shoes was designed using engineering bionic technology. The bionic cushioning unit was then processed based on the bionic design model, and the shoe soles were tested with six impact energies ranging from 3.3 J to 11.6 J for a drop hammer impact and compared with the conventional control sole of the same size. The results indicated that the bionic forefoot area absorbed 9.83–34.95% more impact and 10.65–43.84% more energy than the conventional control forefoot area, while the bionic heel area absorbed 26.34–44.29% more impact and 28.1–51.29% more energy than the conventional control heel area when the controlled impact energy varied from 3.3 J to 11.6 J. The cushioning performance of the bionic cushioning sole was generally better than that of the conventional control sole, and the cushioning and energy-absorption performances of the heel bionic cushioning unit were better than those of the forefoot bionic cushioning unit. This study provides innovative reference and research ideas for the design and development of sports shoes with good cushioning performance.

## 1. Introduction

As people attach greater and greater importance to exercise and health, their criterion for selecting footwear has shifted from aesthetic to functional [1]. Cushioning performance is one of the basic functional indicators of shoe soles, which not only plays a crucial role in the design and development of sports shoes, but also is an important factor in the evaluation of wearing comfort [2,3]. The sole of a shoe consists of insole, midsole, and outsole, among which midsole is the core of its cushioning technology. How to reasonably configure the material and structure of the midsole has become a major concern in the cushioning technology of shoe soles [4,5,6]. In fact, modern midsoles are generally made of foam-like materials processed to produce resistance at the foot–ground interface through the compression and elasticity of the material, consuming the mechanical energy generated by collision, thus playing a role in reducing impact load [7]. Generally speaking, the softer the material and the thicker the midsole, the better the cushioning performance, but accompanied by higher risks of injuries and lower stability [8]. Relying solely on material flexibility is bound to be contradictory in terms of cushioning performance and stability, which makes it an effective means to deal with the limited midsole thickness space, especially the thinner midsole space of the front foot from the perspective of structural and material coupling [9,10,11]. At the moment, major manufacturers primarily focus on aesthetics while ignoring functionality, and existing research focuses on the heel region, with targeted materials and structural configurations in the forefoot region and heel region, while there is little research on the improvement of the cushioning performance of the sole [12,13].

Improving the cushioning performance of sports shoes based on the engineering bionic principle has gradually attracted attention and begun to be applied in the current structural cushioning technology of sports shoes [14]. Traditional sports shoes limit the release of energy stored in the Achilles tendon and calf muscle during lift-off, resulting in a long stride, which is the cause of excessive braking force and impact force [15,16,17,18]. The African ostrich is known for its heavy-duty, high-speed, and long-lasting athletic characteristics. Adult African ostriches can sustain a speed of about 55 km/h for more than 30 min while supporting their own weight of up to 150 kg [19]. Existing research on the cushioning characteristics of the ostrich foot mainly focuses on the foot pad and metatarsophalangeal joint. S.A.A. El-Gendy et al. found through anatomical studies that the ostrich foot pad was able to effectively absorb the impact forces during exercise and played a cushioning role thanks to the unique structure formed by plantar dermis [20]. Zhang et al. constructed a finite-element 3D model of the ostrich foot using reverse engineering technology and experimentally simulated the plantar pressure distribution of the ostrich foot. Their findings indicated that the pressure on the middle part of toe III of the ostrich foot was the minimum [21]. Wang Haitao from Jilin University extracted the structure of the ostrich foot pad and established a finite-element analysis model accordingly. The modal analysis results suggested that the foot pad was mainly deformed in the form of bending at both ends, which was beneficial to protect the ostrich foot musculoskeletal system from impact damage [22]. When studying the mechanism of metatarsophalangeal joint, they also found that the ostrich metatarsophalangeal joint not only achieved compression and cushioning, but also provided energy return for forward motion [23]. Studies have shown that the metatarsophalangeal joint absorbs 63.3% of the total energy stored in the entire ostrich leg and plays an important role in cushioning and shock absorption [24]. Prof. Rui Zhang et al. studied the mechanism of the ostrich’s sand crossing and found that the metatarsophalangeal joint could promote the rebound of the toe joint during the stirrups off the sand phase when the ostrich was running fast [25]. By observing and analyzing the microstructure of tendons, Zhang et al. suggested that the large number of collagen fibrous structures of tendons worked with the bones of the metatarsophalangeal joint for force transfer and play a role in the energy storage and cushioning of the metatarsophalangeal joint [26].

The research objective of this paper is to develop a sports shoe with excellent cushioning performance by coupling material and structural design in the limited thickness space of the midsole (including forefoot and heel) of sports shoes using engineering bionic technology. Inspired by the excellent cushioning and shock absorption characteristics of the ostrich foot, a new type of bionic cushioning unit for the sole was developed based on the mechanism of the toe pad and the metatarsophalangeal joint, with a targeted functional bionic design for forefoot and heel areas. The cushioning performance of the bionic sole was evaluated and analyzed by testing the cushioning performance of the sole. Therefore, this paper provides innovative reference and research ideas for the design and development of sports shoes with good cushioning performance.

## 2. Materials and Methods

### 2.1. Analysis of Key Bionic Parts of Ostrich Foot

#### 2.1.1. The Skeletal–Tendon of the Ostrich Foot Metatarsophalangeal Joint

**Figure 1:**
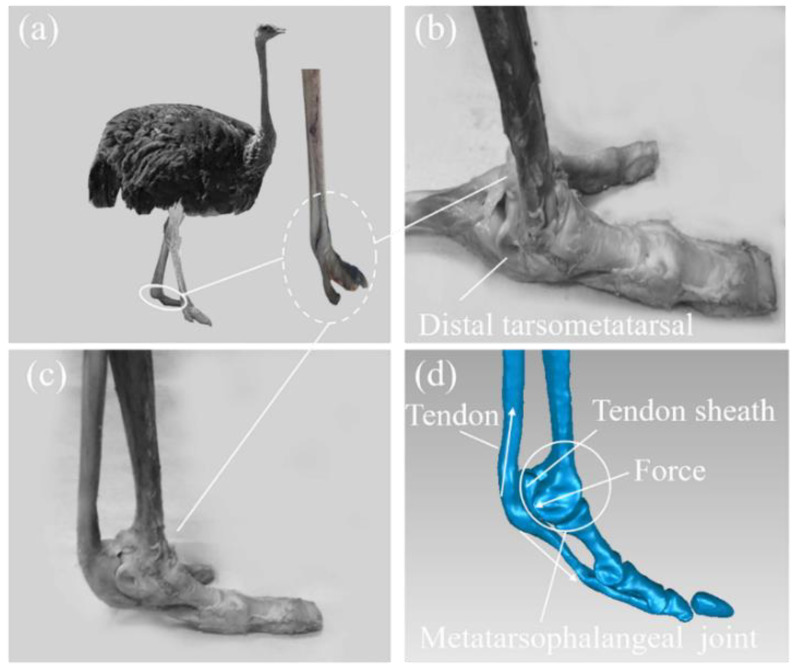
Schematic diagram of the transmission of force in the metatarsophalangeal joint. (**a**) Ostrich foot; (**b**) Metatarsophalangeal joint after fat clearance and tendon separation; (**c**) Metatarsophalangeal joint after fat clearance and tendon recovery; (**d**) 3D model of ostrich foot.

The metatarsophalangeal joint of the ostrich foot remains off the ground throughout locomotion and exhibits an up-and-down floating compression and rebound posture during foot contact, similar to a spring [27]. The model of the skeletal–tendon at the metatarsophalangeal joint of the ostrich foot was reconstructed in reverse by medical imaging scanning, and the schematic diagram of the transmission of the metatarsophalangeal joint forces was analyzed according to the inverse reconstruction model and the anatomical model. It can be seen from the structural features inside the ostrich foot in Figure 1 that the tendon was below the skeletal and extended upward from the tip of the foot in parallel with the skeletal, forming a structural combination of tendon, tendon sheath, and bone joint features at the metatarsophalangeal joint. The presence of tendon restricted the flipping of the metatarsophalangeal joint and kept it in an elevated position. The metatarsophalangeal joint area rotated and was pressed downward during movement, transferring force to the tendon below the joint through tendon sheath. In order to maintain joint elevation, downward pressure was converted into tension force within the tendon. According to studies, at the position of the metatarsophalangeal joint, the stiffness at both ends of the tendon is less than that in the middle, giving the tendon elastic deformation capacity that facilitates the absorption of impact energy by stretching the tendon when the joint is subjected to downward force. Therefore, the tendon plays a cushioning and shock-absorbing role in the metatarsophalangeal joint [28].

**Figure 2:**
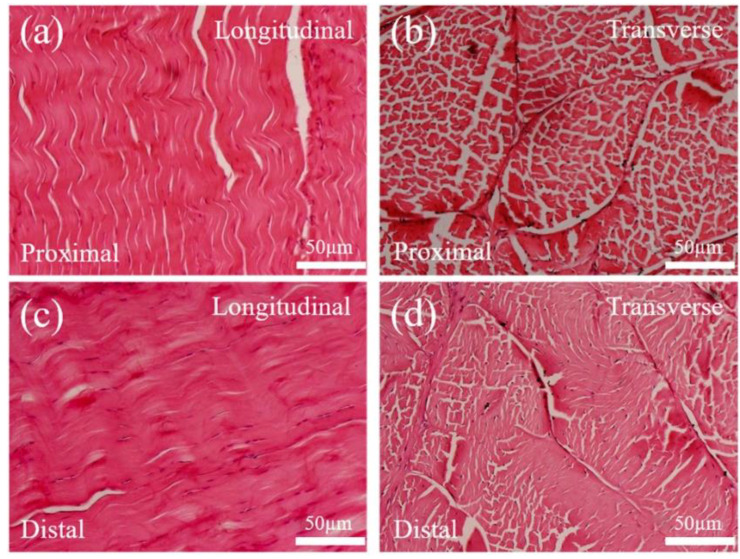
Longitudinal (**a**) and transverse (**b**) views of the third toe tendon at the superior end of the metatarsophalangeal joint.and Longitudinal (**c**) and transverse (**d**) views of the third toe tendon at the lower end of the metatarsophalangeal joint.

We observed tissue sections of the tendons in the metatarsophalangeal joint area to further verify their elastic deformability, as illustrated in Figure 2. In addition, the properties of the material from the tissue structure of the tendon biomaterial were analyzed. The results showed that collagen fibers at the proximal and distal locations of the metatarsophalangeal joint (the less stiff part of the material) were arranged in a wavy pattern in parallel in the longitudinal direction of the tendon. The wavy collagen fibers endowed the tendon with longitudinal tensile deformation and enabled it to convert the kinetic energy of the joint into the elastic potential energy of the tendon by elastic deformation under joint pressure [29].

According to the above analysis, the ability of the metatarsophalangeal joint of the ostrich foot to perform its cushioning function depended on its structure and form of material assembly. During movement, the metatarsophalangeal joint transferred force through the structural assembly of hard bone and soft tendon on the one hand and absorbed impact kinetic energy by taking advantage of the tendon’s elastic deformation on the other.

#### 2.1.2. Pad–Fascia of Ostrich Toe

**Figure 3:**
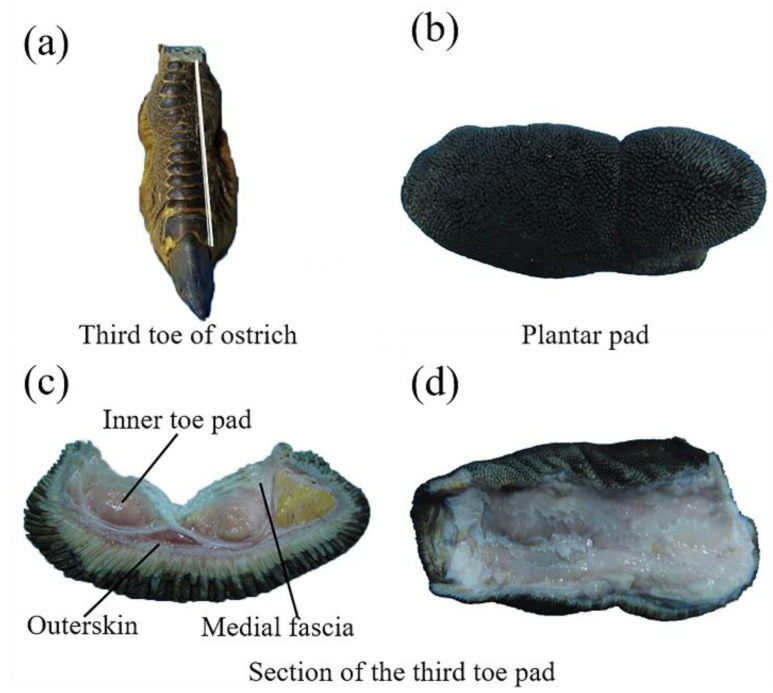
Anatomical diagram of the toes. (**a**) Anatomy of ostrich toes; (**b**)Topography of toe pad bottom surface; (**c**) Section of the third toe pad; (**d**) Overall overhead view.

The third toe of the foot with a relatively wide foot pad was selected as the anatomical sample, and the tissue structure of the foot pad was separated using gross anatomical methods. The sample shown in Figure 3 was extracted from the right foot of male ostriches aged 2 years in the ostrich captive colony site in Team 7 at Qingfeng Farm, Hulin City, Heilongjiang Province, China. In order to avoid damage to the overall structure of the foot pad, a cutting process was performed at the dorsal skin of the toe by cutting the skin layer along the position marked in white with medical scissors, and the skin from the bone at the skin break was slowly peeled to remove the bone and toenail using a medical scalpel. By observing the dissected toe pads and testing the material hardness, we found that the foot pad consisted of three sections, namely, the outer layer of skin, the inner layer of toe pads, and the middle layer of fascia. The footbed shows the structure and material assembly characteristics of the outer layer covering the inner layer and the material hardness from the outer layer to the inner layer from hard to soft.

**Figure 4:**
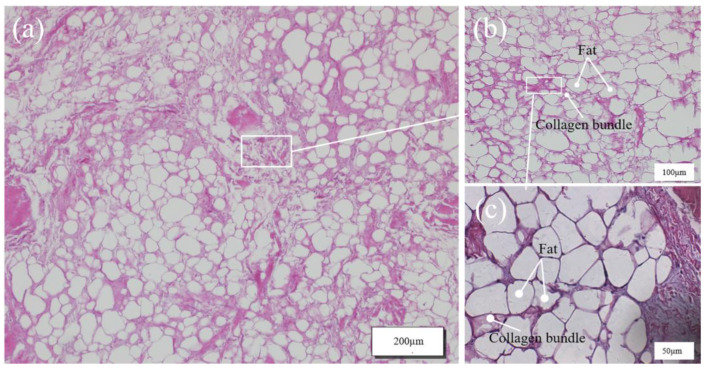
Diagram of a toe pad tissue section. (**a**) Longitudinal section of toe pad; (**b**) Enlarged view of the white box in figure (**a**); (**c**) Enlarged view of the white box in figure (**b**).

In addition, the microstructural information of the toe pad and the interface between the toe pad and the fascia was obtained by tissue section observation, and the microstructures of the two biomaterials were compared to analyze the functional roles of the two materials. As can be seen in Figure 4, the fat in the stained state of the toe pad is marked in white, and a large amount of fat inside was separated into numerous individual small compartments by pink collagen fibers. Fat can play a good role in energy absorption and protect the body from mechanical damage. There was a large amount of fat in the toe pad, forming numerous small, energy-absorbing cushioning units. The microstructure of the toe pad was similar to that of the microporous cushioning foam used in the midsole of sports shoes. Numerous hollow micropores emerged inside microporous foam material in the foaming process, and then gas in the holes was compressed and absorbed impact energy by taking advantage of the material’s deformation caused by force.

**Figure 5:**
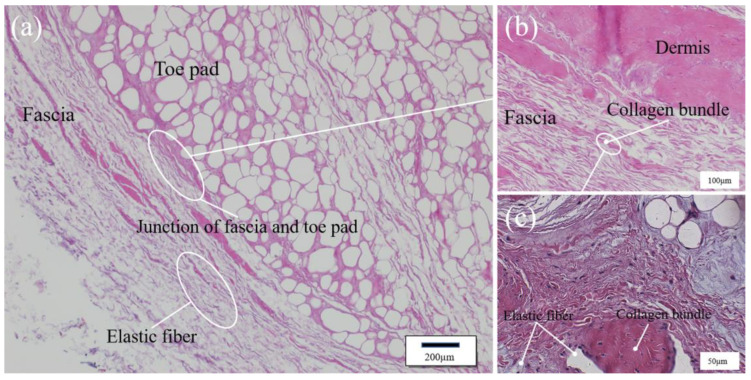
Diagram of a fascia tissue section. (**a**) Longitudinal section of fascia; (**b**) Enlarged view of the oval white box in figure (**a**); (**c**) Enlarged view of the oval white box in figure (**b**).

The fascia is embodied as a septum between the skin and the toe pad, enveloping the outer layer of the toe pad, and its microscopic organization is significantly different from that of the toe pad, which contains numerous fatty compartments, as shown in Figure 5. As can be seen from the figure, stained elastic fibers exhibited a translucent wavy structure, and the fascia consisted of a large number of elastic fibers, among which a small number of collagen fibers were distributed. According to the difference in the tissue structure of the interface biomaterials between fascia and toe pad, the two biomaterials were speculated to have different functions. The wavy elastic fibers were arranged in parallel into fascia so that the fascia, a biological material, have a certain elastic expansion capacity. The elastic wrapping effect of fascia on the toe pad could ensure that the toe pad could maintain a stable shape during deformation and energy absorption.

### 2.2. Bionic Design

**Figure 6:**
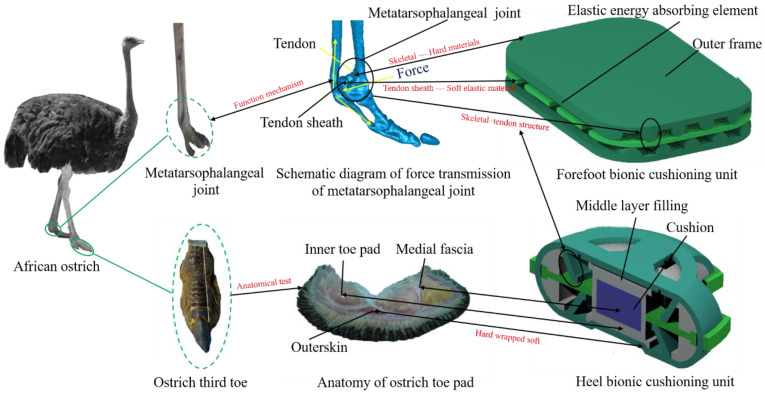
Schematic diagram of forefoot and heel cushioning unit model structure.

Based on the skeletal–tendon structure and material assembly characteristics of the ostrich metatarsophalangeal joint, the forefoot cushioning unit model was designed by replacing the bone with a rigid material to transfer the force to a soft elastic material. The forefoot cushioning unit, as shown in Figure 6, was composed of an outer frame and sandwiched elastic energy-absorbing elements. The inner part of the outer frame was arranged in parallel with a semi-circular convex structure, and the upper frame and the lower frame with a convex structure were staggered. Each individual semi-cylindrical projection interacted with interlayer elastic energy-absorbing element to form a skeletal joint–tendon bionic shock-absorbing structure. When the unit was impacted, the semi-cylindrical projections of the outer frame transferred force to the sandwich elastic energy-absorbing element when the unit was subjected to impact and then converted the kinetic energy of the impact into its own elastic potential energy by means of the elastic deformation of the elastic energy-absorbing element itself.

In order to improve the cushioning performance of the heel, a bionic cushion assembled by the toe pad and fascia inside the foot pad was added on the basis of forefoot cushioning unit. The heel bionic cushioning unit model consisted of an outer frame, an elastic energy-absorbing element, a cushion, and a middle layer filling, whose structural features are shown in Figure 6. The figure shows that the outer frame of hard material was distributed with an extended rocker structure with an included angle of 45° with the frame plate surface in four directions. The extended rocker was staggered up and down, transferring force to the soft, elastic energy-absorbing element placed between the upper and lower rocker as a bionic cushioning structure. The cushion pad was made from the inner layer material (purple part in Figure 6) selected from the traditional microporous foam material of the midsole to replace the fat compartment material of the toe pad, and the outer layer elastically wrapped around the inner layer and integrated with the unit’s interior as a one-piece structure. The same silicon rubber material (white part in Figure 6) was selected for processing. The cushion through the inner layer of deformation energy absorption and the outer layer of elastic wrapping to achieve the toe pad–fascia cushioning function bionic and skeletal–tendon design ideas combined, applied to the heel bionic cushioning unit prototype design.

### 2.3. Sample Preparation

#### 2.3.1. Principles of Sample Size Design

**Figure 7:**
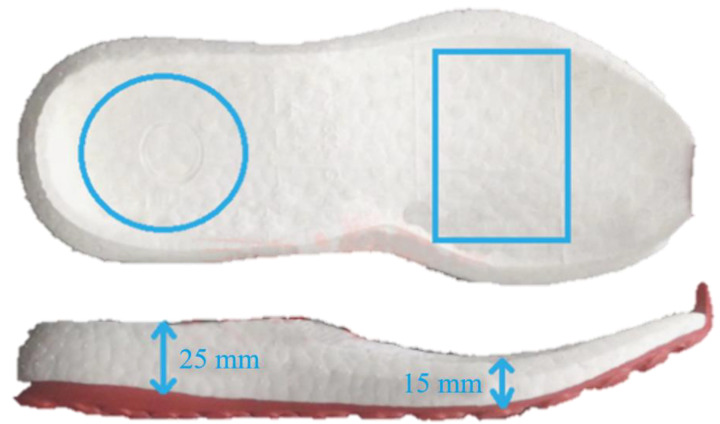
The profile of shoe base.

Taking the traditional foam sole as the base, the cushioning unit was embedded inside the midsole to design and produce physical bionic cushioning unit. The contour size of the bionic cushioning unit was designed with reference to the pre-selected basal to ensure that the unit was fully embedded inside the sole. As shown in Figure 7, the cushioning unit was arranged in the rectangular and circular areas at the forefoot and heel, respectively, whose contour size did not exceed that of the outer edge of the basal. Due to the inconsistent thickness between different areas of the midsole, the thickness of the forefoot and heel cushioning units should not exceed 25 mm and 15 mm, respectively.

#### 2.3.2. The Machining Process of the Bionic Cushioning Unit

In order to better process the physical parts of the cushioning unit and reflect the excellence of structural and material coupling bionic designs, the cushioning unit parts were assembled and processed with traditional conventional materials, and then the bionic cushioning shoe sole unit was physically produced. The outer frame of the unit should have certain stiffness to ensure the stability of the unit’s structural support. Furthermore, in order to prevent the cushioning unit in the midsole from being too stiff to make the foot uncomfortable, the frame material also needed to have certain elastic deformation capacity. Therefore, relatively soft PU rubber material was selected for producing the raw TPU printing wire material with a stiffness range of 80–95 HA TPU, 3D printing outer frame, and elastic energy-absorbing components.

**Figure 8:**
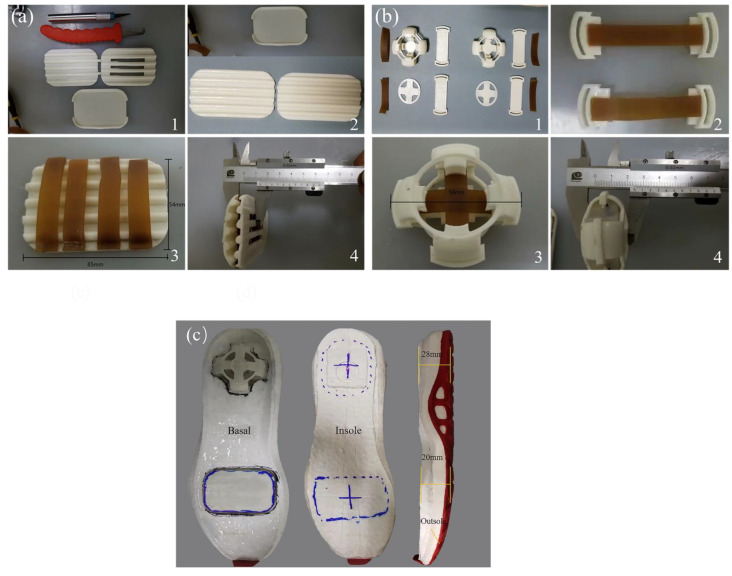
The machining process and complete assembly diagram of cushioning sole. ((**a**) Forefoot cushioning unit. (**b**) Heel cushioning unit. (**c**) The assembly diagram of cushioning sole.)

The outer frame of the forefoot bionic cushioning unit was printed using a 3D printer with silk fusion technology, with a maximum size of 150 × 150 × 150 mm, a nozzle diameter of 0.4 mm, and printing accuracy of 0.1 mm. TPU printing consumables with a diameter of 1.75 mm were selected, and the printhead temperature was set to 230 °C and the hot bed temperature to 30 °C. To avoid plugging in the molding process, the thickness of printing layer was set to 0.2 mm, and printing speed to 40 mm/s. The machining and assembly process of the bionic forefoot cushioning unit is shown in Figure 8a. The entire unit was 12 mm thick, 54 mm wide, and 85 mm long, whose contour dimensions met the dimensional requirements for embedding in the forefoot area, as shown in Figure 8a. The heel bionic cushioning unit frame was processed in the same way. To facilitate the addition of the internal structure of the heel bionic cushioning unit, the outer frame model of heel bionic cushioning unit was split main body and upper cover, which were printed separately. The whole unit was filled with the cushion pad and the outer cushion pad, and the silicone sheet formed with the stiffness of 25–30 HA was purchased for manual cutting. The machining and assembly process of the bionic heel cushioning unit is shown in Figure 8a. The finished heel cushioning unit with a profile diameter of 56 mm and an overall unit thickness of 22 mm is shown in Figure 8b.

The bionic cushioning sole was composed of an outsole, midsole, and insole. With the outsole and midsole as the base, the forefoot and heel units were grooved according to the contour size of the cushioning unit. Then, the cushioning unit was inserted inside the groove hole of the midsole, and the glue particularly used for repairing athletic shoe soles was evenly applied to the upper surface of the midsole and the cushioning unit, and finally to the insole to generate bionic cushioning sole. The thickness of forefoot and heel buffer zones was, respectively, measured to be 20 mm and 28 mm, as shown in Figure 8c. The conventional control sole was produced using a homogeneous material without any structure, which was the same as cushioning unit, and its thickness at the forefoot and heel was the same as that of the bionic cushioning sole.

### 2.4. Impact Test

#### 2.4.1. Test Devices

**Figure 9:**
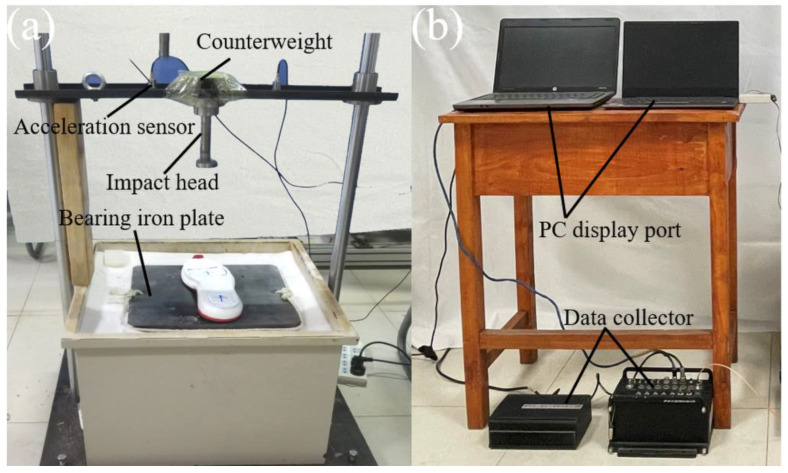
Test device. (**a**) Impact test bench; (**b**) Data acquisition system.

The test device consisted of impact test bench, data collector, and PC data display port, as shown in Figure 9. The impact test bench was composed of impact hammer, light bar, and bearing table. According to the requirements of GB/T 30907, national standard for testing shock absorption in sports shoes, the diameter of the disc where the impact head was in contact with the specimen was 45 mm, and the mass of the impact hammer was 8.5 kg after the addition of the counterweight. The impact head bolted onto the beam was moved vertically up and down along the parallel polished rods during tests.

#### 2.4.2. Test Media

The solid ground was simulated using 45 steel plates in the cushioning performance tests.

#### 2.4.3. Test Process

A total of six high marks were made within the range of 4–14 cm. The impact test acquisition system was connected with the acceleration sensor, and the fastening of the acceleration sensor was checked to avoid the instability of data acquisition caused by loose connection. The connecting part of the polishing rod of the impact hammer and the linear bearing were lubricated with engineering lubricating oil. A height scale of 4 cm was pre-selected to adjust the vertical distance between the impact hammer and the test sole.

The impact test was carried out on the forefoot and heel areas of the bionic cushioned sole and conventional control sole, respectively, at the impact heights of 4 cm, 6 cm, 8 cm, 10 cm, 12 cm, and 14 cm with an impact hammer of 8.5 kg and the impact energies of 3.3 J, 5 J, 6.6 J, 8.33 J, 10 J, and 11.6 J, respectively. The test was repeated at least five times under each impact energy to ensure that all five groups of valid data were recorded.

#### 2.4.4. Data Acquisition and Processing

A data-acquisition and -control system consisting of acceleration sensors (EA-YD-186, 10.07 PC/ms^−2^), a mobile data acquisition device (MDR-81), and a personal computer was employed. The signal was acquired using two acceleration sensors during tests to ensure data accuracy. The acceleration sensors were fastened to the left and right ends of the impact hammer by M5 bolts. The data-acquisition device had a sampling frequency of 256 Hz and an acquisition time of 3 s.

In the software display interface, the data curves were observed to eliminate the data with large variability among the five sets of valid data at each impact energy. Three sets of stable data were exported and saved, each of which contained acceleration data recorded by two sensors. A total of six data samples were retained. The peak negative acceleration values in the data samples were extracted and averaged after the exclusion of the extreme value. Then, the curves were plotted accordingly.

## 3. Results

### 3.1. A Comparative Study of Shoe Sole Cushioning Performance

**Figure 10:**
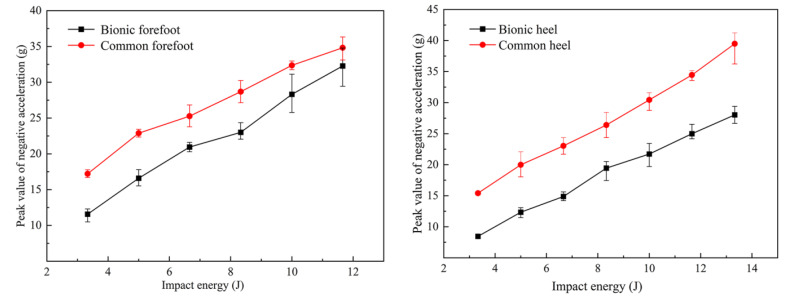
The average negative acceleration peak curve of a bionic or ordinary sole.

The peak negative acceleration curves of the bionic cushioning sole and conventional control sole in the forefoot and heel areas under impact energies of 3.3 J, 5 J, 6.6 J, 8.33 J, 10 J, and 11.6 J are shown in Figure 10. The peak negative acceleration was the maximum acceleration when the impact hammer collided with the sole. The peak negative acceleration of the impact hammer was used to measure the cushioning performance of the sole. When the soles with the same thickness were under the same impact energy, the smaller the negative acceleration peak value obtained on the impact hammer, the better the cushioning performance of the sole. The peak negative acceleration is represented by the g value in the plot and calculated as the ratio of the acceleration value of impact hammer to the gravitational acceleration.

As can be seen from Figure 10, with an increase in impact energy, the peak negative acceleration in the forefoot and heel areas of the bionic cushioned sole approximately increased linearly but lower than that of the control sole in case of the same impact energy. According to the above analysis, the cushioning performance of bionic soles was effectively improved after the addition of forefoot and heel bionic cushioning units compared with the conventional control soles made of ordinary homogeneous materials.

### 3.2. Analysis of Additional Absorption Impact Increment and Growth Rate

Chiu proposed [30] that the relationship between the peak negative acceleration and the impact energy could be derived by fitting a linear equation. The linear regression equations and coefficients of determination of the equations obtained by fitting the four data curves in Figure 10 are shown in Table 1, where a represents the average value of the peak negative acceleration obtained from the impact hammer and E represents the impact energy acting on the sole of the shoe when the impact hammer falls at a specific height.

The same impact energy was applied to the cushioned sole and conventional control, the difference $\Delta a_{m-n}$ in the negative acceleration peak value represents the impact energy additionally absorbed by the bionic sole, that is, compared with the ordinary sole, the additional impact-absorption increment of the bionic sole was calculated by Formula (1). The additional absorption impact growth rate of the bionic sole relative to that of ordinary sole is expressed by $F_{m-n}$, which can be calculated by Formula (2).

(1) $$\Delta a_{m-n}=a_{m}-a_{n}=(\alpha_{m}-\alpha_{n})E+(\beta_{m}-\beta_{n})$$

(2) $$F_{m-n}=\frac{\Delta a_{m-n}}{a_{m}}$$

where $a_{m}$ and $a_{n}$ are the peak negative acceleration obtained by the collision between impact hammer of ordinary soles and bionic soles; α and β are the linear regression equation coefficient; *E* is the impact energy of impact hammer at a specific height acting on the sole of the shoe.

According to the linear regression equation of impact energy and negative acceleration peak value, the calculation equations of additional absorption impact increment and growth rate in the forefoot and heel areas of the sole were obtained, as shown in Table 2.

**Figure 11:**
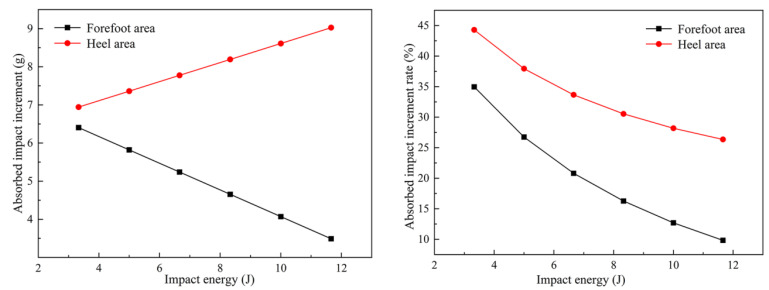
The curve of the bionic sole’s additional absorption impact increment (g) and additional absorption impact growth rate (%).

The six impact energy values were input into formulas to plot the curve, as shown in Figure 11. By comparing the impact-absorption increment in the forefoot and heel areas, we found that additional impact-absorption increment in the heel area was higher than that in the forefoot area under all impact energies. With an increase in impact energy, the impact-absorption increment in the heel area slowly increased, while that in the forefoot area gradually decreased. In the impact energy range of 3.3–11.6 J, the additional absorption impact range in the forefoot and heel areas was 3.48–6.40 g and 6.94–9.02 g, respectively. With an increase in impact energy, the ratio between the two decreased. In the impact energy range, additional impact absorption in the forefoot and heel areas increased by 9.83–34.95% and 26.34–44.29%, respectively.

### 3.3. Analysis of Additional Absorbed Impact Energy Increment and Growth Rate

It was found, from the energy-absorption effect in the forefoot and heel areas relative to the conventional control sole, that the negative acceleration peak of bionic sole was the same as that of ordinary sole, indicating that both have the same cushioning ability, as shown in Formula (3), where $\Delta E_{n-m}$, the difference in the impact energy between the bionic sole and conventional control sole represents the additional energy absorbed by the bionic sole relative to the ordinary sole and can be calculated by Formula (4); $H_{n-m}$, the ratio of the impact energy increment to the initial energy of the impact hammer, is the proportion of the additional energy absorbed by the bionic sole, which can be calculated by Formula (5).

(3) $$a_{m}=\alpha_{m}\times E_{m}+\beta_{m}=\alpha_{n}\times E_{n}+\beta_{n}$$

(4) $$\Delta E_{n-m}=E_{n}-E_{m}=\frac{\left(\alpha_{m}-\alpha_{n}\right)E_{n}+\left(\beta_{m}-\beta_{n}\right)}{\alpha_{m}}$$

(5) $$H_{n-m}=\frac{E_{n}-E_{m}}{E_{n}}=\frac{\left(\alpha_{m}-\alpha_{n}\right)E_{n}+\left(\beta_{m}-\beta_{n}\right)}{\alpha_{m}E_{n}}$$

where $a_{m}$ and $a_{n}$ are the peak negative acceleration obtained by the collision between impact hammer of ordinary soles and bionic soles; α and β are the linear regression equation coefficient; *E* is the impact energy of impact hammer at a specific height acting on the sole of the shoe.

The above formulas were integrated to calculate the additional absorbed energy and additional absorbed energy rate of the soles, forefeet, and heels, as shown in Table 3.

**Figure 12:**
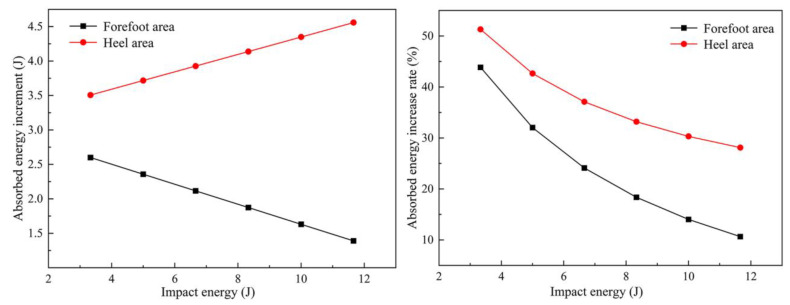
The curve of the bionic sole’s additional absorption impact energy increment (g) and additional absorption impact energy growth rate (%).

The absorbed energy increment of the sole was calculated by the formula in Table 4, and the absorbed energy increment curve of the sole is shown in Figure 12. It can be seen from the curve that as impact energy gradually increased from 3.33 J to 11.46 J, the additional absorbed energy increment in the forefoot area linearly decreased, and the absorbed energy growth rate gradually decreased with an increase in impact energy. The additional absorbed energy was 1.39–2.59 J, and the proportion of additional absorbed energy reached 10.65–43.84%. The energy-absorption increment and the proportion of absorbed energy in the heel area were higher than those in the forefoot area, and the additional absorbed energy increment linearly increased. The proportion of additional absorbed energy gradually decreased with an increase in impact energy. In the entire impact energy range, the additional absorbed energy in the heel area was 3.51–4.56 J, and the proportion of additional absorbed impact energy was 28.1–51.29%.

## 4. Discussion

This study investigated the cushioning effect of bionic soles with bionic cushioning units versus conventional ordinary soles at different impact energies. The results of this study provide ideas for the development of cushioned sports shoes. Our research suggests that the cushioning performance of the bionic cushioned sole in the forefoot and heel areas is better than that of conventional ordinary shoes. At the same impact energy, the bionic cushioning sole absorbs 3.48–6.40 g more impact than the conventional control sole in the forefoot area, with a proportion of additional impact absorption of 9.83–34.95%. The additional absorption impact increment in the heel area is 6.94–9.02 g, and the additional absorption impact growth rate is 26.34–44.29%. Under the same conditions, the heel area outperforms the forefoot area in terms of energy absorption.

We also compared other soles and found that different athletic shoe soles were tested using a single impact energy of 2–10 J in previous studies (Table 4). As different shoes were tested in each study, only the results for the shoes with the best cushioning performance are shown in the table. By comparing the experimental data with those of Yu [30] and Zhou [31], we found that the bionic sole studied in this paper outperformed ordinary soles in terms of both impact absorption and impact energy absorption, which indicates its better cushioning performance. In addition, by comparing the cushioning data obtained at the minimum impact energy in this work with those obtained at the maximum impact energy in the studies of Yu and Zhou, the same conclusion as above can be obtained, so the above analysis results can be ruled out as being caused by the difference in impact energy. The peak negative acceleration is much larger than the other two because the impact energy of this work was much larger than that in the other studies. This study also found that the ratio of impact absorption and energy absorption in the forefoot and heel areas of the bionic sole gradually decreased as the impact energy increased, indicating that the cushioning performance of the bionic sole would be weakened with an increase in impact energy. This is consistent with the findings of the other two studies and proves the validity of this study. In addition, by comparing the data with the studies of Huang [32] and Xiao [33], we found that the maximum peak negative acceleration of this work was the smallest, which also indicated that the bionic sole of this study had better cushioning performance.

Some limitations of this study should be mentioned. Firstly, the bionic cushioning midsole combining the bionic cushioning unit and the midsole matrix of the shoe was not optimized. The coupled cushioning effect of the midsole and the bionic cushioning unit should be considered comprehensively to further investigate the improvement in the cushioning performance of the sports shoes. Secondly, cushioning performance is only one of the functional indicators to measure athletic shoes. On the basis of research on cushioning performance, the balance between multiple performances should be comprehensively considered so to ensure a better cushioning performance and wearing experience from sports shoes. Finally, we have only considered impact tests in this work, and human tests should be added to further increase its credibility.

## 5. Conclusions

Based on the excellent cushioning performance of the ostrich foot and inspired by the structure and material assembly features of the ostrich foot’s metatarsophalangeal skeletal–tendon and the ostrich toe pad–fascia, a functional bionic cushioning unit for the midsole (including the forefoot and heel) area of athletic shoes was designed using engineering bionic technology. The bionic cushioning unit was processed based on the bionic design model, and then shoe soles were tested under impact energies ranging from 3.3 J to 11.6 J for drop hammer impact. Finally, the test results were compared with those on the conventional control sole with the same size. The results show that the cushioning performance of the sole can be effectively improved by adding a bionic cushioning unit inside the midsole. The cushioning performance of the bionic cushioning unit in the heel area has been improved significantly, while that of the bionic cushioning unit in the forefoot area still needs to be further optimized and improved.

## Figures and Tables

**Table 1:** Linear regression equation of mean negative acceleration peak (a) and impact energy (E).

Shoe Sole Area	Shoe Sole Type	
	Bionic	Ordinary
Forefoot	$a_{1}=2.41E+4.03\;(R^{2}=0.99)$	$a_{2}=2.06E+11.46\;(R^{2}=0.99)$
Heel	$a_{3}=1.98E+2.14\;(R^{2}=0.99)$	$a_{4}=2.23E+8.25\;(R^{2}=0.99)$

**Table 2:** Calculation equation for absorption impact (g) and absorption impact growth rate (%) in the sole area.

Shoe Sole Area	Variable	
	Additional Impact Absorption (J)	Percentage of Additional Absorbed Impact (%)
Forefoot	$\Delta a_{2-1}=-0.35E+7.43$	$F_{2-1}=(-0.18E+7.43)/(2.06E+11.46)$
Heel	$\Delta a_{4-3}=0.25E+6.11$	$F_{4-3}=(0.25E+6.11)/(2.23E+8.25)$

**Table 3:** Calculation equation of the additional absorbed impact energy (J) and additional absorbed energy growth rate (%) of the bionic sole.

Shoe Sole Area	Variable	
	Additional Absorbed Energy (J)	Percentage of Additional Absorbed Energy (%)
Forefoot	$\Delta E_{1-2}=(-0.35E_{n}+7.43)/2.06$	$H_{1-2}=(-0.35E_{n}+7.43)/2.06E_{n}$
Heel	$\Delta E_{3-4}=(0.25E_{n}+6.11)/2.23$	$H_{3-4}=(0.25E_{n}+6.11)/2.23E_{n}$

**Table 4:** Comparisons of Impact Testing Methods Used in Previous Studies and in the Present Study.

Investigator	Shoe Sole Area	The Range of Impact Energy (G)	Additional Impact Absorption (g)	Percentage of Additional Absorbed Impact (%)	Additional Absorbed Energy (J)	Percentage of Additional Absorbed Energy (%)	Peak Value of Negative Acceleration (g)
Yu HB [30]	Forefoot	2.35–5.88	0.016–0.019	7.15–12.49	0.396–0.466	7.92–16.88	1.05–2.40
	Heel	2.35–5.88	0.010–0.019	7.06–8.14	0.246–0.447	7.59–10.45	1.10–2.30
Zhou S [31]	Forefoot	2.35–5.88	1.5–5.9	0.221–0.243	0.31–1.22	0.142–0.210	6–16
	Heel	2.35–5.88	1.6–5.3	0.235–0.237	0.33–1.30	0.157–0.210	5–16
Hung HT [32]	Forefoot	-	-	-	-	-	-
	Heel	2–6	-	5.0–7.3	0.52–0.60	12–33	10–17
Xiao Y [33]	Forefoot	-	-	-	-	-	-
	Heel	6–10	-	-	-	-	23–32
Present study	Forefoot	3.3–11.6	3.48–6.40	9.83–34.95	1.39–2.59	10.65–43.84	12–31
	Heel	3.3–11.6	6.94–9.02	26.34–44.29	3.51–4.56	28.10–51.29	6–27.50

## Data Availability

Data are contained within the article.

## References

[1] Lieberman D.E., Venkadesan M., Werbel W.A., Daoud A.I., D’Andrea S., Davis I.S., Mang’Eni R.O., Pitsiladis Y. (2010). Foot strike patterns and collision forces in habitually barefoot versus shod runners. Nature.

[2] Lin S., Song Y., Cen X., Bálint K., Fekete G., Sun D. (2022). The Implications of Sports Biomechanics Studies on the Research and Development of Running Shoes: A Systematic Review. Bioengineering.

[3] Li J., Gu Y., Lu Y., Wang Y. (2009). Biomechanical study on the core technology of sports shoes. China Sport Sci..

[4] Ruder M., Atimetin P., Futrell E., Davis I. (2015). Effect of Highly Cushioned Shoes on Ground Reaction Forces during Running. Med. Sci. Sports Exerc..

[5] Chu H., Lin P. (2007). Friction Analysis of the Sneakers Insole. J. Biomech..

[6] Ma R., Lam W.-K., Ding R., Yang F., Qu F. (2022). Effects of Shoe Midfoot Bending Stiffness on Multi-Segment Foot Kinematics and Ground Reaction Force during Heel-Toe Running. Bioengineering.

[7] Hoogkamer W., Kipp S., Frank J.H., Farina E.M., Luo G., Kram R. (2017). A Comparison of the Energetic Cost of Running in Marathon Racing Shoes. Sports Med..

[8] Robbins S., Waked E., Gouw G.J., McClaran J. (1994). Athletic footwear affects balance in men. Br. J. Sports Med..

[9] Clarke T., Frederick E., Cooper L. (1983). Effects of Shoe Cushioning Upon Ground Reaction Forces in Running. Int. J. Sports Med..

[10] Hsu C.-Y., Wang C.-S., Lin K.-W., Chien M.-J., Wei S.-H., Chen C.-S. (2022). Biomechanical Analysis of the FlatFoot with Different 3D-Printed Insoles on the Lower Extremities. Bioengineering.

[11] Prajapati M.J., Kumar A., Lin S.C., Jeng J.Y. (2022). Multi-material additive manufacturing with lightweight closed-cell foam-filled lattice structures for enhanced mechanical and functional properties. Addit. Manuf..

[12] Liu L. (2015). Study on the Effect of Sole Shock Absorption Structure on Foot Shock Absorption System. Ph.D. Thesis.

[13] Cui L.N., Zhang X., Shi J. Research on functional shoe soles. Proceedings of the 6th Seminar on Functional Textiles and Nanotechnology Applications.

[14] Cheng P., Qu F. Overview of the progress of sports shoes technology. Proceedings of the 12th National Sports Biomechanics Academic Exchange Conference.

[15] Delgado T.L., Kubera-Shelton E., Robb R.R., Hickman R., Wallmann H.W., Dufek J.S. (2013). Effects of Foot Strike on Low Back Posture, Shock Attenuation, and Comfort in Running. Med. Sci. Sports Exerc..

[16] Hill C.M., DeBusk H., Knight A.C., Chander H. (2020). Military-Type Workload and Footwear Alter Lower Extremity Muscle Activity during Unilateral Static Balance: Implications for Tactical Athletic Footwear Design. Sports.

[17] Heiderscheit B.C., Chumanov E.S., Michalski M.P., Wille C.M., Ryan M.B. (2011). Effects of Step Rate Manipulation on Joint Mechanics during Running. Med. Sci. Sports Exerc..

[18] Perl D.P., Daoud A.I., Lieberman D.E. (2012). Effects of Footwear and Strike Type on Running Economy. Med. Sci. Sports Exerc..

[19] Alexander R.M., Maloiy G.M.O., Njau R., Jayes A.S. (1979). Mechanics of running of the ostrich (*Struthio camelus*). J. Zool..

[20] El-Gendy S.A.A., Derbalah A., Abu El-Magd M.E.R. (2012). Macro-microscopic study on the toepad of ostrich (*Struthio camelus*). Veter-Res. Commun..

[21] Zhang R., Wang H., Zeng G., Li J. (2016). Finite element modeling and analysis in locomotion system of ostrich (*Struthio camelus*) foot. Biomed. Res. India.

[22] Zhang R., Han D., Yu G., Wang H., Liu H., Yu H., Li J. (2020). Bionic research on spikes based on the tractive characteristics of ostrich foot toenail. Simulation.

[23] Schaller N., D’Août K., Herkner B., Aerts P. (2007). Phalangeal load and pressure distribution in walking and running ostriches (Struthio camelus). Comp. Biochem. Physiol. Part A Mol. Integr. Physiol..

[24] Rubenson J., Lloyd D.G., Heliams D.B., Besier T.F., Fournier P.A. (2010). Adaptations for economical bipedal running: The effect of limb structure on three-dimensional joint mechanics. J. R. Soc. Interface.

[25] Zhang R., Zhang S.H., Liu F. Analysis of the mechanism of trans-sand movement in ostriches. Proceedings of the Innovating Agricultural Engineering Science and Technology to Promote the Development of Modern Agriculture—2011 Annual Academic Conference of Chinese Society of Agricultural Engineering.

[26] Zhang R., Han D., Luo G., Ling L., Li G., Ji Q., Li J. (2017). Macroscopic and microscopic analyses in flexor tendons of the tarsometatarso-phalangeal joint of ostrich (*Struthio camelus*) foot with energy storage and shock absorption. J. Morphol..

[27] Zhang S., Clowers K., Kohstall C., Yu Y.-J. (2005). Effects of Various Midsole Densities of Basketball Shoes on Impact Attenuation during Landing Activities. J. Appl. Biomech..

[28] Luo G. (2016). Bionic Research on Vibration Damping Adaptive Walking Foot Based on Structural Characteristics of Ostrich Metatar-Sophalangeal Joint.

[29] Lieberman D.E., Warrener A.G., Wang J., Castillo E.R. (2015). Effects of stride frequency and foot position at landing on braking force, hip torque, impact peak force and the metabolic cost of running in humans. J. Exp. Biol..

[30] Yu H.-B., Zhang R., Yu G.-L., Wang H.-T., Wang D.-C., Tai W.-H., Huang J.-L. (2021). A New Inspiration in Bionic Shock Absorption Midsole Design and Engineering. Appl. Sci..

[31] Zhou S. (2020). Bionic Design and Analysis of Football Soles. Comput. Digit. Eng..

[32] Chiu H.-T., Shiang T.-Y. (2007). Effects of Insoles and Additional Shock Absorption Foam on the Cushioning Properties of Sport Shoes. J. Appl. Biomech..

[33] Xiao Y., Hu D., Zhang Z., Pei B., Wu X., Lin P. (2022). A 3D-Printed Sole Design Bioinspired by Cat Paw Pad and Triply Periodic Minimal Surface for Improving Paratrooper Landing Protection. Polymers.

